# Low Compliance to Handwashing Program and High Nosocomial Infection in a Brazilian Hospital

**DOI:** 10.1155/2012/579681

**Published:** 2012-06-06

**Authors:** Lizandra Ferreira de Almeida e Borges, Lilian Alves Rocha, Maria José Nunes, Paulo Pinto Gontijo Filho

**Affiliations:** ^1^Departamento de Imunologia, Microbiologia, Parasitologia e Patologia, Instituto de Patologia Tropical e Saúde Pública, Universidade Federal de Goiás, Goiânia, GO, Brazil; ^2^Programa de Pós Graduação em Imunologia e Parasitologia Aplicadas, Universidade Federal de Uberlândia, Uberlândia, MG, Brazil; ^3^Faculdade de Medicina, Comissão de Controle de Infecção Hospitalar, Universidade Federal de Uberlândia, Uberlândia, MG, Brazil; ^4^Instituto de Ciências Biomédicas, Comissão de Controle de Infecção Hospitalar, Universidade Federal de Uberlândia, Uberlândia, MG, Brazil

## Abstract

*Background*. It is a fact that hand hygiene prevents nosocomial infection, but compliance with recommended instructions is commonly poor. The purpose of this study was to implement a hand hygiene program for increase compliance with hand hygiene and its relationship with nosocomial infection (NI) and MRSA infection/colonization rates. *Methods*. Compliance to hand hygiene was evaluated in a hospital by direct observation and measured of health care-associated infections, including methicillin resistant *Staphylococcus aureus*, before and after an educational intervention, using visual poster, colorful stamps, and feedback of the results. *Results*. Overall compliance did not increase during intervention, only handwashing before and after patient contact has improved from 40% to 76% (*P* = 0.01) for HCWs, but NI and MRSA rates remained high and stable. *Conclusion*. In a combination of high prevalence of NI and low compliance to hand hygiene, the programme of measure does not motivate the HCW hand hygiene. Future interventions should employ incremental evaluation to develop effective hand hygiene initiatives.

## 1. Introduction

Hand hygiene is the single most important measure of prevention and control of nosocomial infection and can significantly reduce the burden of disease, in particular in developing countries [1, 2]. Unfortunately, compliance with recommended hand hygiene procedures has been unacceptably poor, with mean baseline rates of 5% to 81% [3–7].

The identification of several risk factors associated with poor hand hygiene compliance is of extreme importance in the design of an education programme [4]. On the other hand, both nosocomial infection and colonization by methicillin resistant *Staphylococcus aureus* (MRSA) have become increasingly common during the past two decades [8], especially in countries with limited resources [9].

The hand hygiene campaign at the University of Geneva Hospital was the first which reported experience of improvement hand hygiene compliance and reduction nosocomial infection and MRSA transmission [4]. The purpose of the present study was implementation of the program for increase hand hygiene compliance and its association with nosocomial infection (NI), MRSA infection, and colonization rates.

## 2. Methods

### 2.1. Study Design

This study was developed in four different wards clinical, surgical, pediatric, and adult medical-surgical intensive care unit (ICU) in a teaching hospital in Brazil, under 12 months, after approval by the ethics committee of the institution.

### 2.2. Data Collection

Two observers were trained to conduct the prevalence of nosocomial infection, MRSA screening of patients, evaluation of hand hygiene adherence, and feedback of results.

Nosocomial infection (NI) was identified and definite according to Centers for Disease Control and Prevention (CDC), and asymptomatic catheter-associated urinary tract infection by urine culture positive with ≥10^5^ CFU/mL.

Surveillance of MRSA colonization was assessed for nasal culture from with swab and inoculating in Manitol Salt Agar, incubated at 35°C for 48 hours. Colonies that were identified as *Staphylococcus aureus* were screened for methicillin resistance in Muller-Hinton agar supplemented with 4.5% sodium chloride and 6 *μ*g/mL of oxacillin, according to CLSI [10].

Hand hygiene compliance with procedures was measured using methods based on Pittet et al. [4]. Observation of health care workers in patient care were performed during morning and afternoon, and compliance were defined as hand hygiene practice before and after any contact with a patient or with the inanimate material inside the patient's room [11].

Data on handwashing compliance including unit, shift, sex, category of HCWs, and activities classified according to their risk of cross-infection [11]: high risk (before patient contact or/between a dirty and a clean site on the patient), medium risk (after contact with patient or body fluid or after patient care) and low-risk (activity involving indirect patient contact or hospital maintenance).

During regular meetings, in half of the studies (two times per ward) with a multidisciplinary group of HCWs, were presentations of hand hygiene rates displayed, and feedback data. Color posters that emphasized the importance of hand hygiene, and performance feedback, were used to help the intervention and some individual bottles of alcohol handrub were distributed.

### 2.3. Statistical Analysis

Proportions were compared by using Chi-square tests or Fisher's exact test and McNemar to compliance and Student's test for continuous variables. It was considered statistical significance when *P* values were less than 0.05, using GraphPad Prism version 4.0 for Windows (San Diego, CA, USA), Epi Info version 5 (Atlanta, USA), and BioEstat 5.0 (Belem, Para, Brazil) for these calculations.

## 3. Results

### 3.1. Before-Intervention

In 52 sessions of observation, 119 opportunities for hand hygiene were collected. The average compliance was 21.0% (Table 1), all using water and soap. Hand hygiene with alcohol solution was observed once opportunity after handwashing. Hand hygiene compliance was statistically significant among health care workers and was lower in surgical and clinical wards, among doctors, during morning and in procedures associated with a low-risk for transmission (not showed).

The rate of nosocomial infection was 28.9%, especially in the pediatric (31.6%) and critical (53.3%) ward. The most frequent infections were urinary tract infection (17; 30.4%) and surgical-site infection (15; 26.8%) (Table 2). The length mean of stay was 42.9 days to develop NI (range 2–80) and the uses of the urinary catheter and antibiotic were major risk factors to NI (*P* < 0.05, not showed). *S. aureus* was detected in 25% patients, including colonized (19%) and infected (6%). Methicillin sensitive *S. aureus *(MSSA) was isolated from 38 (15%) and MRSA from 25 (10%) of them.

### 3.2. After-Intervention

**Figure 1:**
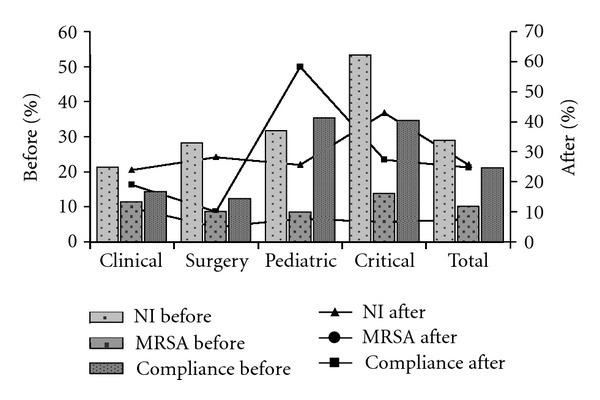
Epidemiological indicators distribution and compliance to hand hygiene in pre and post intervention in the Brazilian hospital. The bars represent the assessments in the period before the intervention and the lines after. NI: nosocomial infection; MRSA: methicillin resistant *Staphylococcus aureus. *

The compliance was similar (24.8%) compared with previous period. Although, adherence was highest in nurses 83.3% and with increase in the frequency of the hand hygiene before and after (*P* = 0.05) (Table 1). The nosocomial infections were 25.7% (Table 2), as after the feedback, decrease in infection and colonization of *S. aureus* rates was 4.1% and 13%, respectively (Figure 1), with the prevalence always greater of MSSA to MRSA but not significantly, even for the length of stay in hospital (46.9 days).

## 4. Discussion

Hand hygiene remains one single and most effective means to prevent, control, and reduce healthcare-associated infections [12, 13]. Based on clinical, experimental, and epidemiological studies, the handwashing and the use of the alcohol-based solutions are strongly recommended, according to the CDC 1A and 1B [12]. But, compliance to recommendations permanence low in worldwide, among HCWs, was with an overall of about 40% [12]. Despite the compelling scientific evidence that hands are the most important vehicle for transmission of nosocomial pathogens [2, 14], we observed in our study a disapproving 25% of compliance hand hygiene, with different levels between hospitals wards, with the pediatric a little higher (58%).

In an observational study, Pittet and colleagues [4] measured the rates of compliance hand hygiene before and during implementation of a program of hand hygiene improvement in Geneva, Switzerland. This hospital-wide program resulted in an increase in the rate of compliance from 48% to 66% over a three-year period and significant decreases in the number of hospital acquired infections from 29% to 17% and MRSA carrier or attack rate of MRSA [15]. Our study, MRSA and MSSA colonization exhibited small variation (12–32%), most significant in critical unit and the proportion of colonization was always higher than infection.

Most infection control programs in developing countries with limited resources are understaffed and handwashing depends mostly in having soap, towels, and sinks available [16]. Poor compliance with hand hygiene is common among HCWs [2] elsewhere factors associated with them include heavy workloads, performing activities with cross-transmission, glove use, discourage, and accessibility to physical structure [4, 17]. We observed the same problems as lack of infrastructure in some units, as sinks difficult location and empty alcohol gel dispensers.

The effective measure to improve hand hygiene compliance has been routine observation and feedback [18]. Our intervention hand hygiene was the primary focus of the investigation targeted the importance of hand transmission nosocomial infection, in principle using the poster campaign and feedback. After intervention, the rates of HI and infection/colonization by MRSA and compliance to hand hygiene have not varied significantly, without important changes. Unlike Pittet et al. [4], based on a poster campaign together with a generalized promotion of alcoholic handrub as an alternative of soap and water handwashing, reduced the nosocomial infection rate and MRSA transmission.

Overall compliance remained stable, in our study (21% and 25%) differently of achieved by Pittet et al. [4] (48% and 66%) that associated with alcoholic rub substantially increase it. Handrub offer the advantage of being less time consuming, probably a factor influencing compliance, especially in demanding situation [19]. In addition, hand hygiene improved significantly among nurses, because they presented more opportunities for hand hygiene, according with other studies [4, 7, 20].

Handrubbing with alcohol-based solution is more effective than handwashing for the decontamination of HCWs hands, besides less irritation of hands [2]. Pittet et al. [4] reported that hand disinfection substantially increased compliance, while handwashing with soap and water remained stable.

Lately, the multimodal/bundle improvement strategy that led to success of the campaign included repeated monitoring of compliance and hand hygiene performance feedback, communication and education tools, constant reminders in the work environment, active participation and feedback at both the individual and organizational levels, involvement of institutional leaders, besides measuring control of HI specifics [2, 12].

This study attempted to investigate an intervention in less time by introducing with alcohol gel, but several investigators reported improved adherence after implementing various interventions, therefore short follow-up periods did not confirm behavioral improvements [12].

Until now, the best scientific evidence of the effectiveness of multimodal intervention strategies in infection control is from studies conducted in developed countries only [21], but in setting with limited resources, as public Brazilian hospitals, compliance with recommendation with hand hygiene by HCWs is very low as shown in this study, with rates of hospital infection remained high, even after the intervention, as pointing out that hand hygiene was poor even though that they were being observed. According to more recent evidence, interventions previously thought to be ineffective such as education are modestly successful [22]. Interpersonal factors are individual characteristics that influence behavior such as knowledge, attitudes, beliefs, and personality traits [23].

Observed HCWs that had a trend to recontaminate their hands and touching other objects, during the patient's care and not handwashing after removal of the gloves. In our view this is the first that study evaluates the impact of a campaign to promote: hand hygiene in the rates of nosocomial infection and infection/colonization by *S. aureus *in a hospital in Brazil, as a whole, and we know that were there some time and the limitations mostly lack of accreditation for HCW.

In conclusion, as mentioned by Sax et al. [24], efforts to improve hand hygiene practices of HCWs have already traveled far over the past few years, by the application of human factors engineering, how alcohol-based hand rubbing as quicker and more effective method, when compared to handwashing, and mainly its location at the point of care, and knowledge and education, but this does not motivate our HCWs, as we observed in our study. Cultural and behavior issues a complex and must be considered to explain the poor compliance.

Implementing hand hygiene to prevent healthcare associated infection has been proven to be a highly cost effective intervention in industrialized countries but our results suggest that the strategy to obtain an improvement in compliance with hand hygiene in developing countries is a hard task, because the risk of acquiring nosocomial infection is increasing.

## Figures and Tables

**Table 1:** Characteristics of opportunities for hand hygiene and compliance before and after intervention.

Variables	Before intervention *n* (%)	After intervention *n* (%)	*P* ^#^
Hand hygiene opportunities	119	117	
Overall compliance	25 (21.0)	29 (24.8)	0.68
Before procedure	4 (16.0)	1 (3.4)	0.37
After procedure	10 (40.0)	6 (20.7)	0.45
Both (before and after)	10 (40.0)	22 (75.9)	0.05*
Handwashing	20 (100.0)	16 (88.9)	—
Alcohol handrub	1 (5.0)	4 (22.2)	0.37
Glove use	9 (45.0)	11 (61.1)	0.82
Nurse	9 (45.0)	15 (83.3)	0.30
Physician	7 (35.0)	1 (5.6)	0.07
Other	4 (20.0)	2 (11.1)	0.68
High risk cross-infection	7 (35.0)	7 (38.9)	0.78
Intermediate risk	12 (60.0)	10 (55.6)	0.83
Low risk	1 (5.0)	1 (5.6)	0.47

^#^McNemar; *statistically significant.

**Table 2:** Frequency of nosocomial infection, infection/colonization by MRSA before, and after intervention. OR = odds ratio and CI = confidence interval.

Variables	Before intervention *n* (%)	After intervention *n* (%)	*P* ^#^	OR (CI 95%)
Nosocomial infection	56 (28.9)	44 (25.7)	0.58	1.2 (0.7–1.9)
RTI^1^ lower	9 (16.1)	16 (36.4)	0.03*	0.3 (0.1–0.9)
Surgical-site infection	15 (26.8)	6 (13.6)	0.17	2.3 (0.7–7.5)
Bloodstream infection	9 (16.1)	14 (31.8)	0.10	0.4 (0.1–1.2)
Urinary tract infection	17 (30.4)	12 (27.3)	0.90	1.2 (0.4–3.0)
Others^2^	9 (16.1)	7 (16.0)	0.80	1.0 (0.3–3.4)
Use of ≥2 antibiotics	22 (39.3)	21 (47.7)	0.52	0.7 (0.3–1.7)
Exposure to ≥3 devices	9 (16.1)	10 (22.7)	0.55	0.7 (0.2–1.9)
*S. aureus* infection	15 (6.0)	6 (4.1)	0.58	1.5 (0.5–4.4)
MRSA^3^ infection	10 (66.7)	5 (83.3)	0.62	0.4 (0.01–5.8)
*S. aureus* colonization	48 (19.0)	19 (13.0)	0.16	1.6 (0.9–2.9)
MRSA colonization	15 (31.3)	6 (31.6)	0.79	0.9 (0.3–3.6)

^1^RTI: respiratory tract infections; ^2^conjunctivitis, meningitis and/or skin and eye infection; ^3^MRSA: methicillin resistant *Staphylococcus aureus*; ^#^chi-square; *statistically significant.
